# TNFa alter cholesterol metabolism in human macrophages via PKC-θ-dependent pathway

**DOI:** 10.1186/1471-2091-14-20

**Published:** 2013-08-03

**Authors:** A Zhi Sha Ma, Qian Zhang, Zhi Yuan Song

**Affiliations:** 1 Department of Cardiology, Southwest Hospital, The Third Military Medical University, Chongqing, China; 2 Department of Cardiology, The Fifth Hospital of PLA, Yinchuan, China

**Keywords:** Reverse cholesterol transport, Cholesterol efflux, TNFa

## Abstract

**Background:**

Studies have shown that inflammation promoted atherosclerotic progression; however, it remains unclear whether inflammation promoted atherosclerotic progression properties by altering cholesterol metabolism in human macrophages. In the present study, we evaluated a potential mechanism of inflammation on atherogenic effects. We evaluated the ability of TNFa to affect Reverse cholesterol transport (RCT) and cholesterol uptake and its mechanism(s) of action in human macrophages.

**Results:**

We initially determined the potential effects of TNFa on cholesterol efflux in the human macrophages. We also determined alterations in mRNA and protein levels of ABCA1, ABCG1, LXRa, CD-36, SR-A in human macrophages using quantitative real-time polymerase chain reaction (PCR) and Western immunoblot analyses. The cholesterol efflux rate and protein expression of ABCA1, ABCG1, LXRa, CD-36, SR-A were quantified in human macrophages under PKC-θ inhibition using PKC-θ siRNA. Our results showed that TNFa inhibited the rate of cholesterol efflux and down-regulation the expression levels of ABCA1, ABCG1 and LXRa and up-regulation the expression levels of CD-36, SR-A in human macrophages; PKC-θ inhibition by PKC-θ siRNA attenuated the effect of TNFa on ABCA1, ABCG1, LXRa, SR-A, CD-36 expression.

**Conclusions:**

Our results suggest TNFa alter cholesterol metabolism in human macrophages through the inhibition of Reverse cholesterol transport and enhancing cholesterol uptake via PKC-θ-dependent pathway, implicating a potential mechanism of inflammation on atherogenic effects.

## Background

Atherosclerosis is a chronic inflammatory disease characterized by early and prolonged presence of macrophages within the innermost layer of the arterial wall [1]. Arterial-wall macrophages uncontrolled uptake of modified low-density lipoprotein (LDL) and caused foam-cell formation, resulting in the development of atherosclerosis [2]. Three main mechanisms are known to be anti-atherosclerotic: Endothelial progenitor cells, plaque neovascularization and reverse cholesterol transport (RCT). Recently, RCT has received special attention in the literature. The entire macrophage-specific RCT, regulated predominantly by the macrophage transporters ABCA1, ABCG1 [3], is a process to remove excess cholesterol from peripheral macrophages to HDL and transport them to the liver for subsequent elimination as bile acids and neutral steroids [4].

Tumour necrosis factor alpha (TNFa) is a pro-inflammatory cytokine involved in initiating inflammatory responses [5]. Human studies and animal models implicate TNFa in atherosclerotic plaque formation [6]. TNFa is produced primarily by macrophages, but also by a broad variety of cell types including lymphoid cells, mast cells, endothelial cells; it promotes macrophage activation, lymphocyte trafficking and homeostasis,cellular infiltration of the plaque and stimulates production of other cytokines which increase plaque instability leading to thrombus formation [7]. Cross-sectional data indicate positive associations between TNFa and degree of atherosclerosis or level of prevalent CVD [8]. However, it is still unclear that TNFa promote atherosclerotic plaque formation whether related to alter cholesterol metabolism in human macrophages. Therefore, we set out to investigate the effect of TNFa treatment on Reverse cholesterol transport function and the expression of CD-36, SR-A in human macrophages.

## Methods

### Materials

TNFa (Hoffmann-La Roche Ltd, Basel, Switzerland), Total RNA extraction reagent RNAiso Plus, PrimeScript RT reagent kit, SYBR-Green PCR kit (Takara, Japan). Western immunoblot reagents were purchased from the Beyotime Institute of Biotechnology (China). All other chemicals were of the best grade available from commercial sources.

### Cell culture

Human peripheral blood monocytes were isolated from three healthy volunteers (Lin Wang, Peng Zhou, Rui Ma, Department of Cardiology, Southwest Hospital, The Third Military Medical University, Chongqing, China) using Ficoll/Hypaque gradient centrifugation. The pooled monocytes from the volunteers were incubated in DMEM supplemented with 10% autologous serum for 10 days so that they would differentiate into macrophages. This research carried out on humans was in compliance with the Helsinki Declaration.

### Cellular cholesterol efflux experiments

Human macrophages were cultured as indicated above. Human macrophages were then labeled with ^3^H-cholesterol (0.3 μCi/mL) in serum-free DMEM medium containing 50 μg/ml ox-LDL and 0.2% bovine serum albumin (BSA) for 24 h. The cells were washed twice with phosphate buffered saline (PBS) and incubated in 2 mL of DMEM media containing 0.2% BSA without or with TNFa at 5 ng/ml, 10 ng/ml for 48 h. The media were then replaced with DMEM containing 0.2% BSA in the presence of lipid-free apoA-I (10 μg/mL) or HDL (50 μg/mL) for 24 h. Efflux media was obtained at the times designated and centrifuged to remove floating cells. Monolayers were washed in PBS in duplicate, and cellular lipids were extracted using isopropanol. Media and cell-associated [^3^H] cholesterol were then measured using liquid scintillation counting. Percent efflux was calculated with the following equation: [total media counts/(total cellular counts + total media counts)] × 100%.

### RNA isolation and real-time quantitative polymerase chain reaction analyses

Total RNA was extracted using RNAiso Plus reagent in accordance with manufacturer’s instructions. The PCR primers were synthesized by Shanghai Sangon (Shanghai, China), and the primer sequences used were the following: ABCA1: forward primer: 5′-AAG CCA AGC ATC TTC AG TTC-3′, reverse primer: 5′-CCA TAC AGC AAG AGC AGA AGG-3′; ABCG1: forward primer: 5′-ATA CAG GGG AAA GGT CTC CAA T-3′, reverse primer: 5′-CCC CCG AGG TCT CTC TTA TAG T-3′; LXRa: forward primer: 5′-AGG CCG GTG CTG AGT ATG TC-3′, reverse primer: 5′-GGG CTC CAT AAA GTC ACC AA-3′; CD36: forward primer: 5′-GTC TTC CCA ATA AGC ATG TCT CC-3′, reverse primer: 5′-GTC TTC CCA ATA AGC ATG TCT CC-3′; SR-A: forward primer: 5′-TGA ACG AGA GGA TGC TGA CTG −3′, reverse primer: 5′-TGT CAT TGA ACG TGC GTC AAA-3′; PKC-θ: forward primer: 5′-ACA TCT ACA GTA TAC TCA CAG-3′; reverse primer: 5′-ACT CGT ACT ATG GAC CAC ATC-3′; GAPDH: forward primer: 5′- AGG CCG GTG CTG AGT ATG TC −3′, reverse primer: 5′- TGC CTG CTT CAC CAC CTT CT −3′. Real-time quantitative polymerase chain reaction (PCR) was performed with SYBR® *Premix Ex Taq*™ II, on a Bio-Rad LightCycler with an iQ3.1 realtime PCR system. Melt curve analyses of all real-time PCR products was used to produce a single DNA duplex. Quantitative measurements were obtained using the ∆∆ Ct method, and expression of GAPDH was used as an internal control.

### Western immunoblot analyses

Cells were harvested and protein extracts prepared in accordance with the manufacturer’s instructions. Western immunoblot analyses [12% SDS-PAGE; 30 μg protein per lane] were then performed using rabbit anti-ABCA1, anti-ABCG1, anti-LXRα, anti-CD-36, anti-SR-A and anti-GAPDH (Abcam, USA)-specific antibodies. Proteins were visualized using enhanced chemiluminescence.

### Transfections for LXRa silencing and screening effective PKC-θ siRNA fragments

The PKC-θ-siRNA specific for mouse PKC-θ and nonsilencing (control) siRNAs were synthesized by Shanghai Genechem (Shanghai, China). Human macrophages (1 × 10^6^cells/well) were transfected using Lipofectamine 2000 (Invitrogen). Following 48 h transfection,the second siRNA fragment of PKC-θ suppressed expression of the PKC-θ gene by 75% according to RT-PCR analyses. The oligonucleotide sequences used to construct siRNA used in this study were: 5-ATC TCA ATG ACG CTG AGT T-3 for PKC-θ (PKC-θ-siRNA).

### LXRa siRNA transfection and western immunoblot analyses

Human macrophages were grown in culture flasks at a density of 1 × 10^7^/ml for 12 hours, washed with PBS, and TNFa at 10 ng/ml, 10 ng/ml was added to the DMEM culture media containing 10% human serum. The negative control was added to the first culture flask and PKC-θ-siRNA was added to the remaining flasks and cultured for 96 hours. Cells were harvested and protein extracts were prepared in accordance with the manufacturer’s instructions. The proteins were then subjected to Western immunoblot analyses [12% SDS-PAGE; 60 μg protein per lane] using rabbit anti-ABCA1, anti-ABCG1, anti-LXRα, anti-CD36, anti-SR-A and anti-GAPDH (Abcam, USA)-specific antibodies. Proteins were visualized using enhanced chemiluminescence methods.

### Statistical analyses

Data are expressed as mean ± standard error of the mean (SEM). Results were analyzed using ANOVA with SPSS 13.0 software. P < 0.05 was considered statistically significant. All experiments were performed in at least triplicate.

## Results

### TNFa inhibited cholesterol efflux in human macrophages

**Figure 1:**
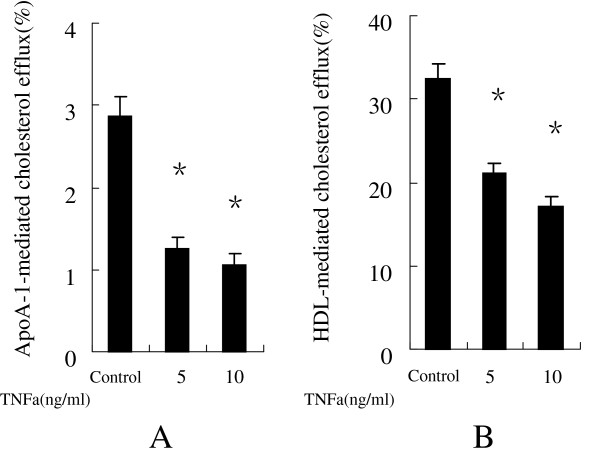
**TNFa inhibit cholesterol efflux from human macrophages.** After human macrophages were labeled with 3H-cholesterol, the indicated doses of either TNFa or the vehicle were added to the cultures, which were then incubated in the presence of either 10 μg/mL human apoA-I **(A)** or 50 μg/mL of human HDL **(B)** for 24 h. Cholesterol efflux was determined as described in Methods. Data are presented as mean ± SEM, *P < 0.05 vs control.

We initially examined the effect of TNFa on cholesterol efflux in human macrophages using liquid scintillation counting assays. Figure 1(A, B) shows that TNFa at 5 ng/ml, 10 ng/ml obvious inhibited apoA-I- and HDL-mediated cholesterol efflux in human macrophages compared with the negative control.

### TNFa down-regulated the mRNA and protein expression of ABCA1, ABCG1, LXRa in human macrophages

**Figure 2:**
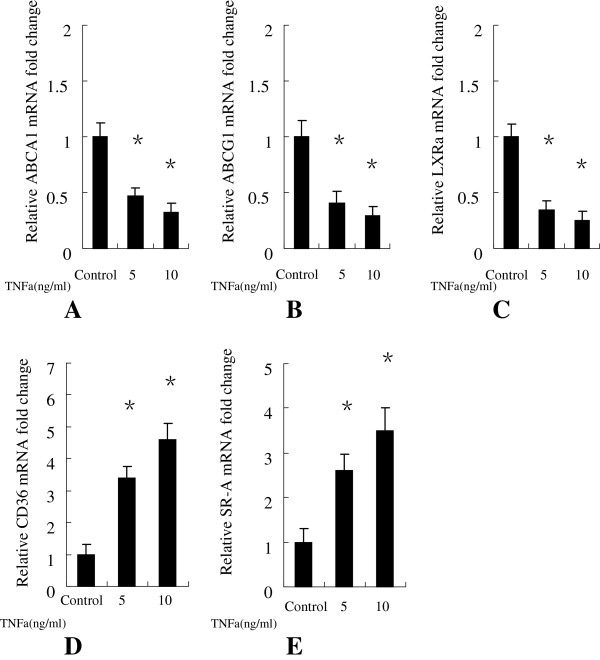
**Human macrophages were treated either with or without TNFa at 5 ng/ml, 10 ng/ml for 24 h. (A)** ABCA1, **(B)** ABCG1, **(C)** LXRa,**(D)** CD-36, **(E)** SR-A gene expression were measured using real-time quantitative PCR. Similar results were obtained in three independent experiments. Data are mean ± SEM. *P < 0.05 vs control.

**Figure 3:**
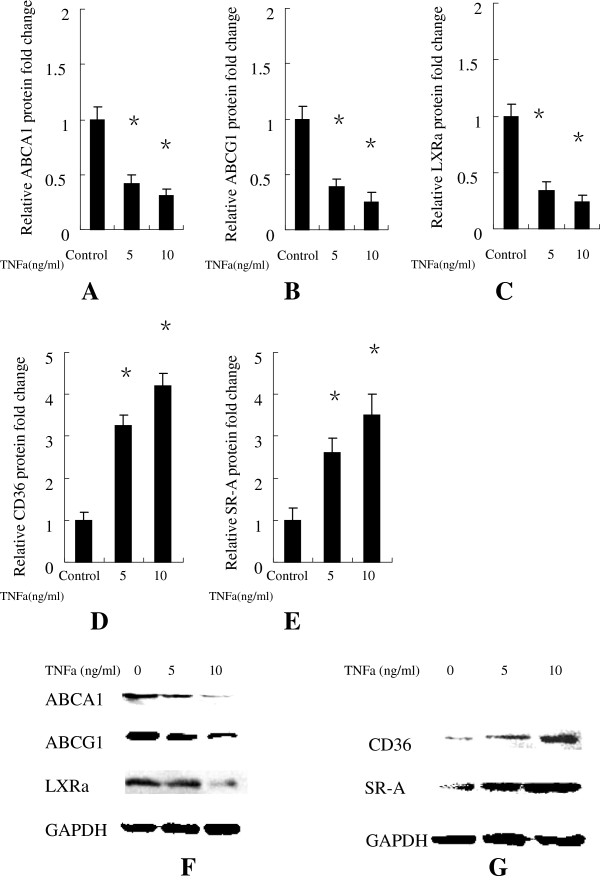
**Human macrophages were treated without or with TNFa at at 5 ng/ml, 10 ng/ml for 48 h. (A)** The Relative protein fold change of ABCA1; **(B)** The Relative protein fold change of ABCG1; **(C)** The Relative protein fold change of LXRa; **(D)** The Relative protein fold change of CD-36; **(E)** The Relative protein fold change of SR-A. **(F)** ABCA1, ABCG1, LXRa and **(G)** CD-36, SR-A protein expression were measured using Western immunoblotting techniques. Similar results were obtained in three independent experiments. Data are mean ± SEM. *P < 0.05 vs control.

To further explore the effects of TNFa on human marcophage-mediated RCT, we examined the effects of TNFa on ABCA1, ABCG1 and LXRa expression in human macrophages using real-time quantitative PCR and Western immunoblot analyses. Our results showed that TNFa at 5 ng/ml, 10 ng/ml significantly decreased the mRNA (Figure 2) and protein (Figure 3A, B) expression levels of ABCA1, ABCG1, LXRa.

### TNFa up-regulated the mRNA and protein expression of CD-36, SR-a in human macrophages

We then examined the effects of TNFa on the expression levels of CD-36, SR-A expression in human macrophages. As demonstrated in Figure 2 that the mRNA expression levels of CD-36, SR-A were significantly up-regulated in human macrophages treated with TNFa at 5 ng/ml, 10 ng/ml compared with the negative control. The protein expression of CD-36, SR-A were also significantly up-regulated (Figure 3A, C) in human macrophages treated with TNFa at 5 ng/ml, 10 ng/ml compared with the negative control.

### PKC-θ inhibition by siRNA significantly attenuated the effect of TNFa on ABCA1, ABCG1, LXRa, CD-36, SR-a expression

**Figure 4:**
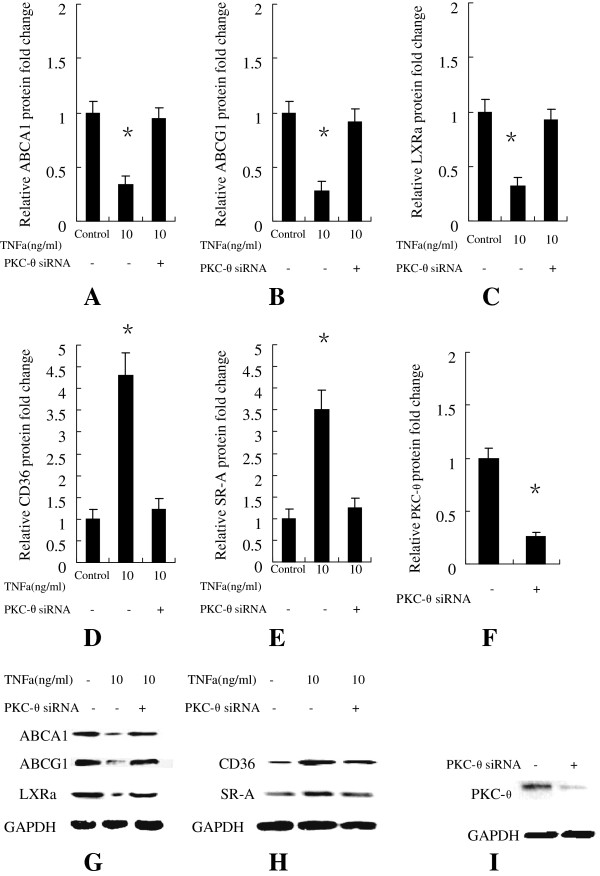
**Human macrophages were transfected with either the negative control or PKC-θ-siRNA and then incubated without or with TNFa at 10 ng/ml, 10 ng/ml for 96 h.** The Relative protein fold change of **(A)** ABCA1, **(B)** ABCG1, **(C)** LXRa **(D**) CD-36, **(E)** SR-A were shown in Figure 4. **(G)** ABCA1, ABCG1, LXRa and **(H)** CD-36, SR-A protein expression were measured using Western immunoblotting techniques. Human macrophages were transfected with either the negative control or PKC-θ-siRNA and then incubated for 96 h. **(F)** Relative protein fold change of PKC-θ in human macrophages transfected with either the negative control or PKC-θ-siRNA. **(I)** PKC-θ protein expression were measured using Western immunoblotting techniques. Similar results were obtained in three independent experiments. Data are presented as mean ± SEM, *P < 0.05 vs control.

We next investigated the effect of treatment with PKC-θ siRNA on TNFa (10 ng/ml)-induced expression of ABCA1, ABCG1, LXRa, CD-36, SR-A. PKC-θ siRNA suppressed the expression of PKC-θ gene (Figure 4D) by 76% according to Western immunoblot analyses. As showed in Figure 4(A, B, C ) that PKC-θ siRNA significantly attenuated the effect of TNFa on ABCA1, ABCG1, LXRa, CD-36, SR-A expression.

## Discussion

TNFa plays a central role in inflammation; it induces the expression of other proinflammatory molecules, chemotactic cytokines and adhesion factors [9]. In vivo and in vitro studies have shown that high levels of TNFa lead to exacerbation of the inflammatory response [10]. This, together with its potent immunomodulator activities, has been suggested as important to the pathogenesis of diseases such as asthma and Atherosclerosis [11]. In this study, we evaluated a potential mechanism of TNFa on atherogenic effects. As shown in Figure 1A and B, TNFa at 5 ng/ml, 10 ng/ml significantly inhibited both apoA-I- and HDL-mediated cholesterol efflux from human macrophages. Next, we determined the mRNA and protein levels of ABCA1, ABCG1, LXRa in order to elucidate the molecular mechanisms underlying the inhibited cholesterol efflux in human macrophages due to TNFa.

A previous study noted that ATP binding cassette transporters (ABCA1, ABCG1) and LXRa are the best characterized cellular transporters/receptor involved in macrophage RCT [12]. The initial comparison of macrophage-specific RCT between ABCA1-deficient and wild-type mice showed direct evidence that ABCA1 deficiency reduces RCT from macrophages to feces in vivo [13]. ABCG1 is another ABC transporter that is able to load more cholesterol onto mature HDLs from the peripheral tissues and is important in allowing macrophages to efflux arterial wall cholesterol, eventually preventing atherosclerotic vascular disease [14].

Our results showed that TNFa at 5 ng/ml, 10 ng/ml significantly decreased the mRNA expression levels of ABCA1, ABCG1 and LXRa compared with the control group (Figure 2), resulting in significantly down-regulated protein expression levels in human macrophages (Figure 3A, B). This indicates that TNFa inhibit reverse cholesterol transport in human macrophages via ABCA1 and ABCG1 -mediated pathways.

Interestingly, we also found that the mRNA (Figure 2) and protein (Figure 3A, C) expression levels of pattern-recognition scavenger receptors CD-36 and SR-A, were significantly up-regulated in human macrophages treated with TNFa at 5 ng/ml, 10 ng/ml. SR-A belongs to scavenger receptor A family, and has been implicated in atherosclerosis [15]. CD36 is a member of the class B scavenger receptor family and its activation has been implicated in foam cell formation. It appears that SR-A and CD36 account for greater than 90% of the lipid accumulation in macrophages exposed to oxidized LDL [16]. So it suggested that TNFa can promote cholesterol uptake by enhancing the expression of CD-36 and SR-A in human macrophages.

Protein kinase C (PKC) comprises a family of serine/threonine kinases that are involved in the regulation of many cellular responses, including proliferation, differentiation, stress responses, and lipid metabolism [17]. PKC isozymes have been classified in three subfamilies: conventional (cPKCs α, βI, βII and γ), novel (nPKCs δ, ϵ, θ and η) and atypical (aPKCs λ, ι, μ, ζ). Conventional PKCs are regulated by diacylglycerol (DAG), hosphatidylserine and calcium, whereas novel PKCs are calcium-independent, but regulated by DAG and phosphatidylserine, and atypical PKC isoforms are regulated by phosphatidylserine, but calcium, DAG- and TPA-independent [18]. Previous study showed that PKC activation mediates production of granulocyte/macrophage colony-stimulating factor (GM-CSF), which plays a priming role in Ox-LDL-induced macrophage proliferation [19]. Protein kinase C-β and –δ mediate cholesterol accumulation in PMA-activated macrophages and another report shows that stably overexpressed a dominant-negative of PKC-α inhibits LPS-induced iNOS expression in RAW 264.7 macrophages [20,21]. HIV-1 Tat protein induces TNF-α and IL-10 production via a PKC-βII and -δ isozymes dependent way in human macrophages [22]. 6-Gingerol inhibits ROS and iNOS in lipopolysaccharide-stimulated mouse macrophages,through the suppression of PKC-α and NF-κB pathways, [23].

PKC-θ mediates the critical T cell receptor (TCR) signals selectively required for T cell activation in vivo [24]. PKC-θ plays a crucial role in the activation of various transcription factors such as NF-kB, AP-1 and nuclear factor of activated T cells (NFAT) [25,26]. Mature PKC-θ^−/−^ T cells failed to proliferate and produce interleukin 2 (IL-2) upon TCR stimulation due to defective activation of NF-kB and AP1 [27]. Mice deficient in other isoforms of PKC do not have a defect in T cell activation, thereby reinforcing the importance of PKC-θ in T cell activation [28]. Considering the multiple functions of PKC-θ in T cell activation, it would be interesting to examine whether PKC-θ plays a role in the effect of TNFa on ABCA1, ABCG1, LXRa, SR-A, CD-36 expression in human macrophages.

To confirm whether PKC-θ is involved in the TNFa-induced down-regulation the expression of ABCA1, ABCG1, LXRa and up-regulation the expression of CD-36, SR-A in human macrophages, we utilized PKC-θ-siRNA to inhibit PKC-θ. The results show that PKC-θ-siRNA significantly attenuated the effect of TNFa on ABCA1, ABCG1, LXRa, SR-A, CD-36 expression compared with the negative control (Figure 4A, B, C). This phenomenon confirmed that PKC-θ is involved in the effect of TNFa on ABCA1, ABCG1, LXRa, SR-A, CD-36 expression in human macrophages. These data suggest that PKC-θ activity is involved in the effect of TNFa on ABCA1, ABCG1, LXRa, SR-A, CD-36 expression in human macrophages, and the isotype-selective inhibitor to PKC-θ may suppress atherosclerotic progression.

## Conclusion

These results suggest that TNFa promote atherosclerotic progression by altering cholesterol metabolism though inhibiting Reverse cholesterol transport and enhancing cholesterol uptake in human macrophages via PKC-θ-dependent pathway.

## Competing interests

The authors declare that they have no competing interests.

## Authors’ contributions

AZSM designed the experiments and wrote the manuscript; AZSM carried out cell culture,the molecular genetic studies, the immunoassays and PKC-θ inhibition using PKC-θ siRNA. QZ performed cellular cholesterol efflux experiments and statistical analyses. ZYS participated in study design and coordination and helped to draft the manuscript. All authors read and approved the final manuscript.
